# Methane Quantification Performance of the Quantitative Optical Gas Imaging (QOGI) System Using Single-Blind Controlled Release Assessment

**DOI:** 10.3390/s24134044

**Published:** 2024-06-21

**Authors:** Chiemezie Ilonze, Jiayang (Lyra) Wang, Arvind P. Ravikumar, Daniel Zimmerle

**Affiliations:** 1 Department of Mechanical Engineering, Colorado State University, Fort Collins, CO 80523, USA; chiemezie.ilonze@colostate.edu; 2 Department of Petroleum and Geosystems Engineering, The University of Texas at Austin, Austin, TX 78712, USA; lyrawang06@gmail.com (J.W.); arvind.ravikumar@austin.utexas.edu (A.P.R.); 3 Energy Emissions Data & Modeling Lab, The University of Texas at Austin, Austin, TX 78712, USA; 4 Energy Institute, Colorado State University, Fort Collins, CO 80524, USA

**Keywords:** methane, QOGI, FLIR, emissions quantification, methane quantification, FLIR QL320, Providence Photonics QL320

## Abstract

Quantitative optical gas imaging (QOGI) system can rapidly quantify leaks detected by optical gas imaging (OGI) cameras across the oil and gas supply chain. A comprehensive evaluation of the QOGI system’s quantification capability is needed for the successful adoption of the technology. This study conducted single-blind experiments to examine the quantification performance of the FLIR QL320 QOGI system under near-field conditions at a pseudo-realistic, outdoor, controlled testing facility that mimics upstream and midstream natural gas operations. The study completed 357 individual measurements across 26 controlled releases and 71 camera positions for release rates between 0.1 kg ${Ch}_{4}/h$ and 2.9 kg ${Ch}_{4}/h$ of compressed natural gas (which accounts for more than 90% of typical component-level leaks in several production facilities). The majority (75%) of measurements were within a quantification factor of 3 (quantification error of −67% to 200%) with individual errors between −90% and 831%, which reduced to −79% to +297% when the mean of estimates of the same controlled release from multiple camera positions was considered. Performance improved with increasing release rate, using clear sky as plume background, and at wind speeds ≤1 mph relative to other measurement conditions.

## 1. Introduction

Methane emissions mitigation is a critical element of the global transition to a low-carbon future [1,2,3]. As the major component of natural gas, methane’s global warming potential (GWP) is 84–87 times that of carbon dioxide over a 20-year time scale [4]. Curbing methane emissions is an effective strategy to reduce near-term climate warming, thus allowing a longer time frame to reduce carbon dioxide emissions [5]. The oil and natural gas (O&G) sector is the largest industrial source of methane emissions in the United States, contributing approximately 30% of the total methane emissions in 2021 [6]. In November 2021, the Environmental Protection Agency (EPA) proposed updated rules for methane emissions reduction from the O&G industry [7]. Additionally, starting in 2024, the Inflation Reduction Act (IRA) will impose a methane charge on emissions above a certain threshold at O&G facilities [8]. Thus, accurate quantification of methane emissions is important for effective policy implementation.

To reduce methane emissions, many jurisdictions implement regular leak detection and repair (LDAR) programs at O&G facilities [9,10]. These LDAR programs do not require emissions quantification. One regulatory-approved methodology for emissions detection during LDAR surveys is using handheld, passive, single-channel broadband optical gas imaging (OGI) cameras [11,12]. Passive OGI cameras rely on infrared (IR) imaging of gas species with the most common type operating in the mid-wave spectrum (3 µm–5 µm)—the absorption spectral window of methane and other light hydrocarbons [13,14,15]. LDAR surveys with handheld OGI IR cameras require scanning equipment components at O&G facilities to identify emissions as demonstrated in several field measurement studies [16,17,18,19,20,21,22]. While recent, advanced emissions detection technologies that are based on drones [23,24], automobiles [25,26], and aircraft [27,28] promise faster surveys at a relatively lower cost, they cannot localize emitting components. In addition, they often require days to process emissions data and notify operators of detected emissions [29,30,31]. In contrast, personnel with handheld OGI cameras can detect, localize, and repair emitting sources as soon as they are identified [32,33,34].

Historically, OGI IR cameras did not quantify detected leaks—emissions quantification was performed as an additional measurement step which includes the other test method (OTM) 33A [35,36,37,38,39], downwind tracer flux [16,40,41], and hi-flow sampler (HFS) [16,22,42,43,44,45,46,47,48]. These emission estimation approaches require direct contact with the emission source (i.e., HFS), typically provide aggregate/facility level emission rate (i.e., downwind tracer flux and OTM 33A), or rely on favorable wind transport to perform measurements. For example, the HFS uses attachments (e.g., a sampling hose assembly) to capture and direct gas plumes into the instrument during measurements; hence, successful measurements rely on safe access to the emitting sources. In addition to other operational limitations, sources that are unsafe, inaccessible, or too large for the attachments to cover cannot be quantified by HFS [49,50,51,52].

The quantitative optical gas imaging (QOGI) tool is an add-on device (a tablet) to an OGI IR camera that analyzes plume pixels from videos of hydrocarbon emissions captured by the OGI camera and quantifies emissions using proprietary algorithms [53,54,55]. In summary, for a given IR image of hydrocarbon emission, flow rates are estimated by combining the calculated path concentration (ppm-m) of each pixel from an IR image with assessed plume characteristics (e.g., speed and direction). The difference between the intensity and temperature of the gas plume and the background for each pixel of the plume is evaluated to estimate each pixel’s path concentration [56,57]. The QOGI system (OGI camera + QOGI tablet) is an approved method by the British Columbia Oil and Gas Commission (BCOGC) for comprehensive LDAR surveys [58]. Unlike the HFS, the QOGI system does not require personnel to have physical contact with emission sources to complete measurements. Several manufacturers now offer QOGI systems including handheld and mounted solutions [59,60,61]. Recent measurement studies using the QOGI system have shown poor quantification at very high emission rates [19], identified nonuniform plume background and poor thermal contrast between plume background and emitted gas [17], and external interferences such as noisy heat signatures [62] as some of the limitations inhibiting accurate measurements with the QOGI system. Hence, comprehensive testing is needed to understand the quantification performance of the QOGI system and characterize limiting factors. The system tested in this study is the Teledyne FLIR™ QOGI system (Teledyne FLIR, Wilsonville, OR, USA.), which pairs a QL320™ quantification tablet with a handheld GF320™ OGI IR camera. FLIR™ QL320™ system was selected for testing since at the time of testing, the device was the latest version of Providence Photonics QL100 and QL320 tablets (Providence Photonics LLC, Los Angeles, CA, USA) which have been tested before [53,63,64,65].

The quantification tablets (QL100 and QL320) were originally produced by Providence Photonics and are now offered directly by Teledyne FLIR. In 2015, the Concawe air quality OGI ad hoc group tested the quantification accuracy of the Providence Photonics QL100 QOGI tablet [63]. Three gases (propane, methane, and propylene) were released either individually or mixed in over 61 leak tests, and 31 of them with emissions rates ranging from 10 g/h to 998.7 g/h were quantified and had quantification errors between −23% to 69%. Among the four quantified releases that contained methane (two pure methane releases and two mix releases), the quantification error ranged from −12% to 0% with release rates between 49.7 g/h and 169.7 g/h. However, during the Concawe study, a cool towel was used as a backdrop which provided a more uniform background and enhanced the difference between the apparent temperature of the background and the gas plume temperature (ΔT). Five of the 31 measurements that used a cool towel to enhance thermal contrast had quantification errors ranging from −6% to −23%. Another assessment of the QL100 in 2015 by Abdel-Moati et al. showed an average quantification error of 24% with a standard deviation of 39% for controlled release rates ranging from 54 g ${Ch}_{4}/h$ to 109 g ${Ch}_{4}/h$. When tested on propane, the quantification error ranged from −17% to +43% [53]. The Saskatchewan Research Council (SRC) in 2018 tested the Providence Photonics QL320 tablet over three controlled release rates (1, 5, and 10 L/minute) with poor reproducibility of estimates and errors up to 1570% [64]. The study indicated an error range of ±30% if the temperature contrast between the gas plume and the background is greater than 10 °C over a large number of data sample [64]. Similarly, in 2019, the Alberta Methane Field Challenge (AMFC) project tested the Providence Photonics QL320 tablet (which uses an older version of the quantification algorithm used in FLIR QL320) by conducting about 50 controlled releases ranging from 565 g/h to 36,000 g/h. The study showed an 18% underestimation bias with a linear regression coefficient of 0.82 (0.73, 0.92) over the tested emission rate range [65]. Even though the quantification errors of individual estimates ranged from −90% to 330%, the quantification error when all measurements were aggregated was comparable to that of the HFS [49]. Finally, in 2022, Nagorski Michael, while developing an alternative QOGI algorithm using a single-channel broadband OGI IR camera, tested FLIR’s QL320 over release rates 5 and 10 standard liters per minute (slpm) of methane with a temperature-regulated plume background. The study result showed average quantification errors of −42% and +18% for windspeed categories of 0–0.9 m/s and 0.9–4.5 m/s, respectively, when the release rate was 5 slpm and average errors of −55% and −8% when release rate was 10 slpm. Also, the study did not observe a clear trend in the quantification performance of the QL320 with background temperature so long as there was a sufficient temperature difference between the gas and plume background [14]. In summary, the known existing literature on the QOGI system performance is based on either previous versions of the equipment (hardware and software (Providence Photonics QL320 3.0.0.6)), small sample sizes, or non-robust testing under near-representative field conditions. In addition, a comprehensive and systematic investigation of the impact of measurement factors (wind speed, leak type, distance to emitting source, and plume background) on quantification accuracy and precision of the QOGI system is needed to build confidence in estimates as identified by the Petroleum Technology Alliance of Canada’s Fugitive Emissions Management Program–Effectiveness Assessment study [62]. 

This study presents the first known systematic evaluation of the impact of measurement conditions on the quantification performance of the FLIR QL320 QOGI system under near-field conditions at the Methane Emissions Technology Evaluation Center (METEC), a controlled-test facility which mimics release geometries, rates, and backgrounds encountered at typical O&G facilities. We evaluate the quantification accuracy of individual estimates, as well as the quantification precision when repeated measurements were performed for different camera positions and controlled release rates. We also investigate the impact of the release rate, distance to emitting source, measurement background, leak type, and wind speed on quantification accuracy. 

## 2. Methodology

### 2.1. Testing Facility

The study was conducted at METEC at Colorado State University, Fort Collins, USA. The 8-acre outdoor facility simulates emissions typically associated with upstream and midstream operations. METEC includes non-operational, surface O&G equipment like wellheads, separators, flare stacks, and liquid tanks. About 200 emission sources are strategically located on the equipment such that a wide range of realistic fugitive and vent emissions scenarios can be actualized. Metered natural gas of known composition is transported through buried gas supply tubing from onsite compressed natural gas (CNG) cylinders to the emission points. See Zimmerle et al. [33] for a detailed description of the test facility and the associated gas transport infrastructure for conducting controlled releases.

### 2.2. Experimental Design and Protocol

**Figure 1:**
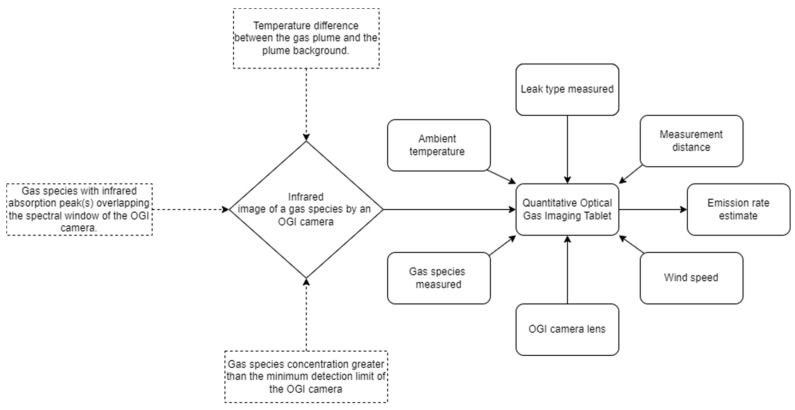
A schematic of the QOGI system (OGI camera and QOGI tablet) that was tested in this study. The diagram elements with broken lines or edges represent the conditions required to visualize gas species with an OGI camera to be quantified. The figure also shows input parameters (setups and measurement conditions) into the QOGI device to generate emission estimates.

Measurements took place from 20 June to 24 June 2022. The QOGI system tested consisted of a FLIR GF320 OGI IR camera and a FLIR QL320 QOGI tablet (henceforth “FLIR tablet”), as illustrated in Figure 1. The software version of the FLIR QL320 was 1.4.1. The Providence Photonics QL320 QOGI tablet (henceforth “legacy tablet”), which operates using an older version (software version 3.0.0.6) of the quantification algorithm used in the FLIR tablet, was used as a backup whenever the FLIR tablet had a low battery. Measurements were performed by a field crew of 2 researchers who operated the equipment and recorded data. One of the field crew members had previously attended the in-person QOGI training sessions provided by Providence Photonics, while the other crew member had previously screened ~40 facilities with the OGI IR camera. The field crew followed the user manual by FLIR when deploying the tablets [66]. The tablets quantify emissions by analyzing the image of the plume captured by the OGI IR camera and operationalizing the tablets for quantification can be carried out either through a “tethered” or “Q-mode” configuration. Under the “tethered” configuration, the camera and the tablet are deployed together in the field and connected with a USB cable such that live feed video from the camera is transferred to the QOGI tablet for quantification while the emission is under observation. Under the “Q-mode” configuration, the OGI camera records emission videos together with required input parameters (e.g., windspeed), while quantification is performed later by analyzing the videos on the QOGI tablet. In this study, emissions were quantified under the “tethered” configuration where possible since it is usually the preferred deployment in field conditions.

**Figure 2:**
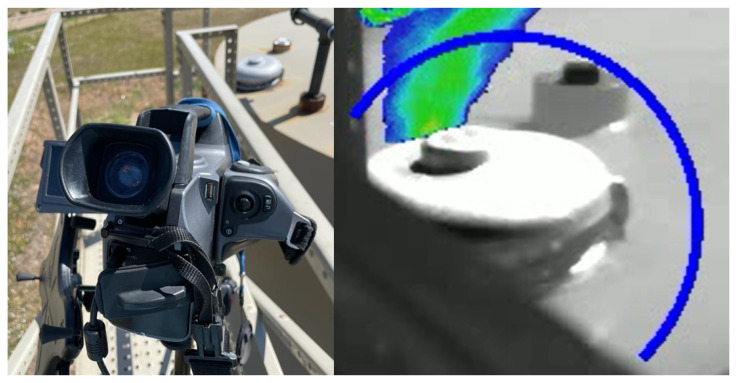
The figure shows a typical deployment of the OGI camera during the study (**left side**) and a snapshot of the infrared image of the observed gas in the QOGI tablet (**right side**). The left side of the figure shows the OGI camera observing emissions from a tank’s thief hatch. The right side of the figure shows colorized enhancements overlaid onto the infrared image of the gas plume being observed (**left side figure**) with a masking feature activated to remove measurement boundaries with visual noise.

This study evaluated only the quantification performance of the QOGI system, i.e., it did not test the detection performance of OGI camera surveys, which is well-known, understood, and available in the peer-reviewed literature [32,33,34]. Hence, this study is constrained by the assumption that the QOGI system is being used to quantify fugitive emissions already identified by an OGI IR camera. The experiments in this study were single-blind: the emission rates of releases conducted by the test center were unknown to the field crew, but they were informed of the leak source. The testing process involved the following steps:

1. The METEC facility operator selected an emission source, initiated a controlled release, waited until the release rate was steady, and then informed the field crew of the location of the emission. The METEC facility operator assigned each experiment a unique numeric identifier (ID) and communicated that (without the emission rate) to the field crew for documentation. An experiment was defined as a controlled release at a given rate flowing through a specified emission point over a given duration.
2. The field crew identified an unobstructed view of the leak source with the gas plume, considering wind direction, measurement distance, and plume background. This was carried out by observing the emission source from multiple perspectives and distances to find the “best view” of the plume (as adjudged by the measurement crew) with enough space to accommodate the camera tripod and camera operator [66]. The field crew mounted the camera on a tripod and positioned it with the emitting source at the center of the measurement boundary as shown on the tablet’s screen (see Figure 2).
3. Parameter data required for quantification were inputted into the tablet which included wind speed (calm (0–1 mph), normal (2–10 mph), or high (>10 mph)), distance to emitting source, leak type (point or diffuse), and ambient temperature. Ambient wind speed and temperature were measured using a handheld digital anemometer, while distance was measured with a measurement tape. While handheld digital anemometers can be highly uncertain and less accurate compared to fixed-mounted 2-D and 3-D anemometers, it is more convenient and faster to use handheld digital anemometers in the field during measurements. Depending on the geometry of an emission point, the field crew selected either “point” (sources with diameter < 2 inches, e.g., connectors, valve packing, etc.) or “diffuse” (sources with diameter > 2 inches, e.g., flanges, thief hatches, etc.) as the leak type on the device before taking measurements. The overlay function was enabled on the tablet to colorize the plume to increase visibility. The field crew also ensured that only the streaming image of the gas plume interacted with the measurement boundary and used the masking feature to remove other areas of visual disturbance (e.g., vegetation on the ground, etc.) when necessary as shown in Figure 2. The field crew selected the viewing angle and distance from the emission source considering the minimum and maximum distance requirement as specified in the manuals for the 23 mm (24° FOV) camera lens—5 and 54 feet from the emission source [66].
4. At each camera position, at least 3 consecutive, individual measurements were taken on the QOGI tablet. A successful measurement was performed when the field crew pressed the tablet’s ‘capture’ button as it turned green indicating a stable measurement condition (measurement opportunity). A stable measurement was defined in the user manual as being when the 10 s quantification result was within 10% of the 1 min quantification result. At least 3 measurements were attempted for each ‘measurement opportunity’ to assess measurement precision without modifying the observation position or allowing environmental conditions to change substantially. For each measurement, the field crew documented the background of the plume (sky, equipment, or ground). In some instances, 3 successful measurements could not be completed from a selected location due to rapidly changing meteorological conditions. At the end of measurements for each camera position, the device was reset ahead of measurements from the next camera position.
5. For each experiment, the field crew attempted measurements from 3 different camera positions by repeating steps 2–4. The controlled release rate during each experiment was approximately the same for all the measurements conducted for an experiment. This represented a simplification of observed field conditions, where temporal variability of emissions has been observed in multiple studies [67,68,69]. Each camera position was assigned a unique ID as no two positions had the same measurement conditions. Approximately 10 min elapsed between camera positions to locate clear views of the plume. Measurement duration varied substantially (a few minutes to >20 min), as in some cases, highly variable meteorological conditions adversely affected measurement efforts. In addition, for a different location, each new camera position had a different measurement distance and/or plume background/perspective. In some instances, fewer than 3 camera positions were identified for an experiment due to obstructions in the field of view or the effect of prevailing unfavorable environmental and/or meteorological conditions (e.g., glints on the plume background, a cloudy sky, etc.).
6. After completing all measurements for an experiment, the field crew notified the METEC facility operator to stop the controlled release to conclude the experiment. The next experiment was then conducted following the same steps either using the same emission source at a different emission rate or an emission source in a different location.

### 2.3. Data Analysis 

Individual measurements (see Supplementary Materials) were identified by the camera position and experiment IDs. Release rates and gas composition data were obtained from METEC release logs at the end of the study. The study team applied response factors for gas species in the controlled release to correctly adjust estimates generated by the QOGI tool (Supplementary Materials Section S1). The field crew attempted 442 measurements, from which 85 measurements were excluded due to missing controlled release data from the test center (27), Q-mode measurements (11), and “wrong” leak type selection (47). Wrong leak type exclusions include cases where emissions from sources like flanges and thief hatch (diameter > 2 inches) were classified as “Point” (diameter < 2 inches) sources instead of “Diffuse” leak type during measurements. While these misclassifications were against the operating protocol, they can happen during active field deployments. Q-mode measurements could not be used due to the unavailability of the QOGI tablets at the end of the study. The 357 valid measurement data were paired with the controlled release data using experiment IDs. Quantification error was assessed for each pair with the 95% confidence interval (CI) on the mean error evaluated as the 2.5 and 97.5 percentiles of the bootstrapped mean errors. Boxplots were primarily used to investigate the impact of the factors (i.e., windspeed, plume background, etc.) by categorizing elements of each factor into groups (e.g., windspeed—calm, normal, and high windspeeds). Since during the measurements, the study team had limited control of the number of sample data points per group, we set a minimum threshold of 30 data points (based on the central limit theorem) as likely being sufficient for statistically significant analysis. Additionally, the Mann–Whitney U and Kolmogorov–Smirnov tests were used to investigate if the error distribution of the groups for each factor investigated was statistically different at a significance level (*p*) of 0.05. 

### 2.4. Study Limitations 

Unlike previous assessment studies captured in the literature, this work systematically evaluates the impact of measurement conditions typically encountered during field deployments and the likely decisions (OGI camera tripod positions) made by field deployment personnel, on the quantification accuracy and precision (estimate reproducibility) of the FLIR QL320 QOGI system. This study also compares measurement scenarios and provides guidance and recommendations on how to use QOGI systems to obtain relatively accurate results in the field. This is important given the fast-growing interest in handheld QOGI systems in the O&G industry because of the popularity of LDAR by OGI camera surveys. Despite these benefits, the study still has limitations as summarized below:

- While METEC mimics real O&G upstream and midstream facilities, not all field conditions were replicated for this study. At METEC, no equipment is heated (which can improve or complicate ΔT) or pressurized (which can cool the plume due to the Joule–Thompson effect at the point of release), which is common for separators (liquid separation equipment) at production facilities. Also, the facility is not characterized by elevated background emissions concentration, equipment vibrations, and noise levels typical in real O&G facilities. All controlled releases were held constant in this study; variable rates are often observed in field conditions, particularly for gas-powered pneumatic controllers.
- OGI cameras are also sensitive to hydrocarbons other than methane that have infrared absorption bands within the spectral range of the camera, particularly ethane and propane. The CNG utilized in this study had a mean gas composition by volume of 84.8% of methane, 8.5% of ethane, 0.7% of propane, and a trace amount of heavier hydrocarbons and other gases. In field conditions, the gas composition varies: upstream (production) emissions contain higher levels of ethane and propane than tested here which increases camera response; however, midstream and downstream emissions may have lower levels of ethane and propane than tested here and hence a lower camera response.
- Field testing took place over 5 days during the summer of 2022 representing a limited range of tested weather conditions. Quantification performance during winter and other associated meteorological conditions were not evaluated. Also, the QOGI system was tested on common components at O&G production facilities and may not represent performance in other sectors of the O&G supply chain.
- Controlled release rates in this study were designed to capture fugitive emission rates observed at production O&G facilities that would be candidates for QOGI quantification. Figure S2 in the Supplementary Materials shows that more than 90% of component-level estimates at production facilities from studies by Allen et al. [16], Bell et al. [41], and Pasci et al. [70] were below the maximum rate conducted in this study (78 slpm of methane or 2.9 kg ${Ch}_{4}/h$). Hence, the study did not assess QOGI performance for larger emitters (up to >10 kg/h) which is an important emission source category at O&G facilities. While the study controlled for the range of emission rates, the distribution of emission rates tested in this study does not represent distributions of emission rates typically obtained at production O&G facilities.
- Finally, prior work on OGI surveys indicated a strong correlation between the experience of the OGI operator and the probability of detecting emissions [33]. Similar dependence may exist in quantification and should be evaluated when broader usage of QOGI would make it possible to statistically sample a range of experience levels in a controlled experiment.

## 3. Results and Discussion

### 3.1. Quantification Accuracy of Individual Measurements

In total, 357 measurements were conducted with the QOGI system across 73 camera positions and 26 experiments. Emissions from four additional experiments could not be quantified from any camera position due to poor imaging background (cloudy sky or weeds on the ground). Experiments had controlled release rates ranging from 2.2 to 88 standard liters per minute (slpm) of CNG (2.0 slpm to 78.0 slpm of methane or 0.07 kg ${Ch}_{4}/h$ to 2.9 kg ${Ch}_{4}/h$) (Supplementary Materials Sections S1.1 and S1.2), and 1 to 11 (mean of 4.9) successful measurements were conducted at each camera position. For each experiment, measurements were taken from one to six camera positions (mean of 2.8) with 4–27 (mean of 13.7) total successful individual estimates. Eight types of emitting sources were used in this study: connector, control box, flange, pressure transducer, pressure release valve (PRV), temperature regulator, thief hatch, and valve packing. Since the legacy tablet was used as a substitute for the FLIR tablet, there was no direct performance comparison between them; however, both tablets produced a similar trend when quantifying controlled release rates within the same range (Supplementary Materials Section S2). The use of the Legacy tablet was limited as much as possible hence accounting for <10% (33 of 357) of all successful measurements. 

**Figure 3:**
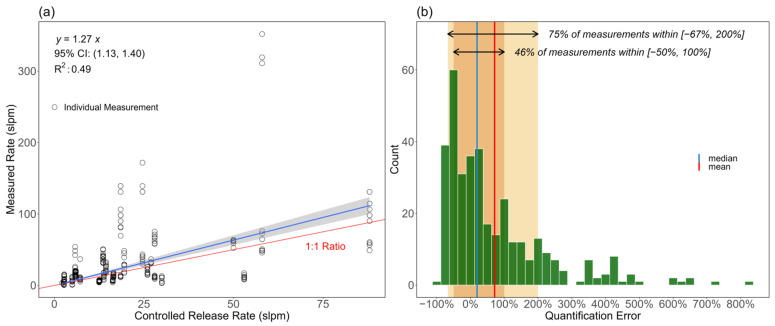
Quantification accuracy of individual estimates: (**a**) measured rates versus controlled release rate and (**b**) distribution of quantification error of individual estimates. In (**a**), the blue line represents linear regression through the origin with the gray shading showing the 95% confidence interval of the regression when bootstrapped. The red line represents the 1:1 ratio, where the measured rate matches the controlled release rate. In both cases, the intercept was forced to zero. In (**b**), the orange shading represents the measured rate within a factor of two of the controlled release rates (−50% to 100% quantification error), and the yellow shading represents the measured rate within a factor of three of the controlled release rates (−67% to 200% quantification error).

Figure 3 examines the quantification accuracy of individual estimates. Figure 3a compares individual rate estimates against controlled release rates. A linear regression analysis with an intercept set to zero indicates a regression coefficient of 1.27 (95% CI [1.13, 1.40])—an overestimation bias of 27%. Across all estimates, individual relative errors ranged from −90% to +831% compared to −90% to +330% from the AMFC study even though the latter tested much larger rates [65]. Results showed that 46% (*N* = 165) of individual estimates were within a quantification factor of 2 (−50% to +100%) of the controlled release rates, while 75% (*N* = 266) of individual estimates were within a factor of 3 (−67% to +200%).

### 3.2. Impact of Quantification Parameters on Accuracy

#### 3.2.1. Emission Rate

**Figure 4:**
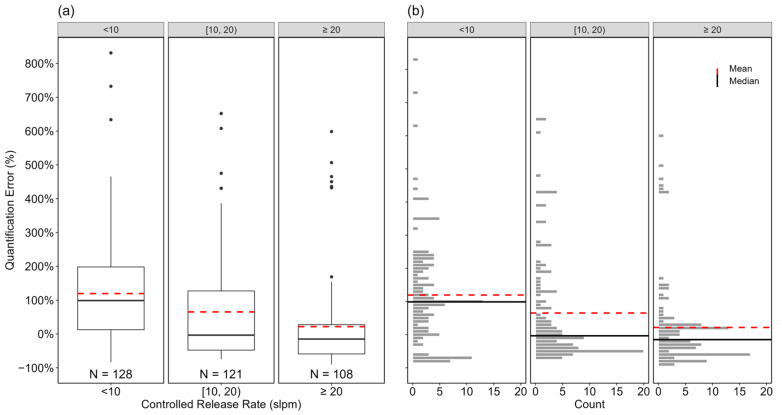
Boxplots (**a**) and distribution (**b**) of the quantification errors of individual estimates based on controlled release rate (<10 slpm, [10, 20) slpm, and ≥20 slpm). The black, circular markers in (**a**) represent outliers as data points outside 1.5 times the interquartile range of the data in each group. The horizontal, black solid, and red dotted lines in both (**a**) and (**b**) indicate the median and mean of the distribution in each group, respectively.. The *x*-axis represents the groups within each parameter, and the *y*-axis shows the quantification error in percentage. The numbers at the bottom of the boxplots represent the sample sizes of individual estimates within each group.

Figure 4 shows the impact of controlled release rate on quantification accuracy. In Figure 4a, the controlled release rates tested were separated into three groups: <10 slpm, [10, 20) slpm, and ≥20 slpm. See Supplementary Materials Section S1.2 for further discussion on the distribution of counts of measurements with the associated controlled release rates tested. The mean and median quantification errors for the groups were +119% (95% CI [+94%, +150%]) and 99%, +65% (95% CI [+40%, +99%]) and −3%, and +22% (95% CI [+0.3%, +53%]) and −15%, respectively. Figure 4b shows the distribution of quantification errors for each controlled release rate range. All three groups had positively skewed (mean > median) distributions that were significantly (statistically using the Mann–Whitney U test) different (*p* < 0.05) with mean errors inflated by outliers as shown in 4a (also see Supplementary Materials Section S3.1). This type of positive skewness has been seen in several other studies of next-generation leak quantification solutions [71,72,73,74]. As controlled release rate increased, there was observed improvement in quantification performance; mean error decreased, the interquartile error range narrowed, and the number and size of outliers dropped. This observed improvement is likely because a large emission rate implies a higher path-integrated concentration, which can increase plume image contrast for the same ΔT and enhance the signal-to-noise ratio for more accurate quantification estimates.

The overestimation bias observed in this study was contrary to the conclusion of the AMFC study which showed 18% underestimation bias (regression coefficient of 0.82 (95% CI [0.73, 0.92])) by the QOGI system tested but agrees with recent studies of other imaging systems that quantified emissions [71,72,73]. The controlled release rate tested in the AMFC study ranged from 15 slpm to 925 slpm, which is about an order of magnitude higher than the rates in our study. A linear regression analysis of overlapping controlled release rates (15 slpm to 88 slpm) from both studies produced coefficients of 1.24 (95% CI [1.05, 1.44]) for our study (*N* = 169) and 1.14 (95% CI [0.72, 1.55]) for the AMFC study (*N* = 32). Although the AMFC study had a smaller sample size and thus a wider confidence interval, the bias from both studies agreed. For rates exceeding the current tested range (>90 slpm), results from the AMFC study showed underestimation bias [65]. In general, results from this study indicate that any single emission rate estimate of emitters routinely identified and fixed during OGI surveys can deviate from the true rates by up to a quantification factor of 10 (×/10 to 10x). However, this uncertainty can be narrowed with more strategic measurement practices discussed below.

#### 3.2.2. Plume Background

A gas plume background with sufficient thermal contrast (ΔT) is needed for successful emission rate estimation with the QOGI system. The QOGI tablets tested in this study required a minimum ΔT of 2 °C for quantification. Since the quantification method of the QOGI tablets tracks changes in the pixel intensity of infrared images, apparent temperature changes or disturbances in the background can interfere with the identification of a plume boundary which can affect quantification performance. These disturbances include shadows, glints, reflections of heat sources on any metallic equipment, and motion such as cloud cover or ground vegetation near the equipment. In this study, plume backgrounds were grouped into three categories, equipment, ground, and sky, as in Zimmerle et al. [33]. To investigate the impact of plume background, the field crew tried to select camera positions with different backgrounds during measurements. A background was classified as “equipment” when the gas plume was viewed against either a different part of the same equipment (e.g., a wellhead casing) or nearby equipment (e.g., a neighboring wellhead unit). A background was classified as “sky” when the plume was viewed against the sky, which may or may not have included cloud cover (e.g., when an elevated emission source like the thief hatch of a tank was viewed against the sky). A background was classified as “ground” when the gas plume was viewed against the ground (i.e., sand, stones, gravel, vegetation). 

**Figure 5:**
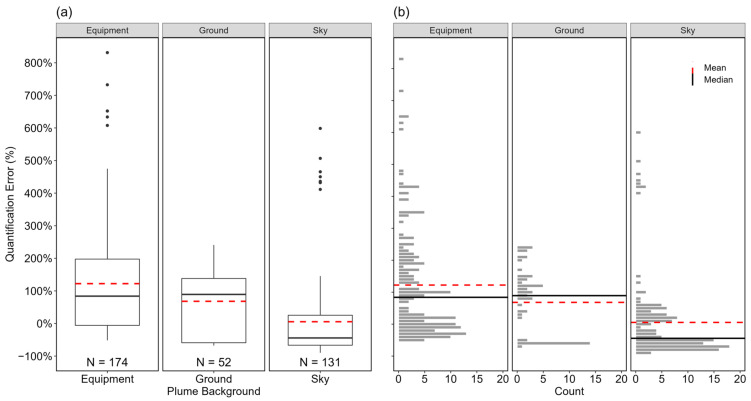
Boxplots (**a**) and distribution (**b**) of the quantification errors of individual estimates based on quantification background (Equipment, Ground, and Sky). The black, circular markers in (**a**) represent outliers as data points outside 1.5 times the interquartile range of the data in each group. The horizontal, black solid, and red dotted lines in both (**a**) and (**b**) indicate the median and mean of the distribution in each group, respectively. The *x*-axis represents the groups within each parameter, and the *y*-axis shows the quantification error in percentage. The numbers at the bottom of the boxplots represent the sample sizes of individual estimates within each group.

Results from the various quantification backgrounds with statistical (using Mann–Whitney U test) different error distributions (*p* < 0.05) are presented in Figure 5. The mean and median quantification errors for ground, equipment, and sky as plume background are +68% (95% CI [+40%, +97%]) and 89%, +122% (95% CI [+98%, +150%]) and 84%, and +5% (95% CI [−13%, +32%]) and −44%, respectively. Some parts of the ground at METEC were covered in vegetation that moved with the wind which made quantification challenging; however, as hinted earlier, reflections and glint from equipment also affected measurements when equipment was the plume background. While a cloudy sky also made quantification challenging, a clear sky was the most favorable background for quantification, noting that the outliers shown in Figure 5a (quantification errors between 400% and 600%) were estimates conducted under high windspeeds (Supplementary Materials Table S4). This result agrees with findings from previous studies and recommendations in the FLIR user manual: clear sky with low apparent temperature provided the best thermal contrast for the tablet’s quantification algorithm [65,66]. 

#### 3.2.3. Windspeed

**Figure 6:**
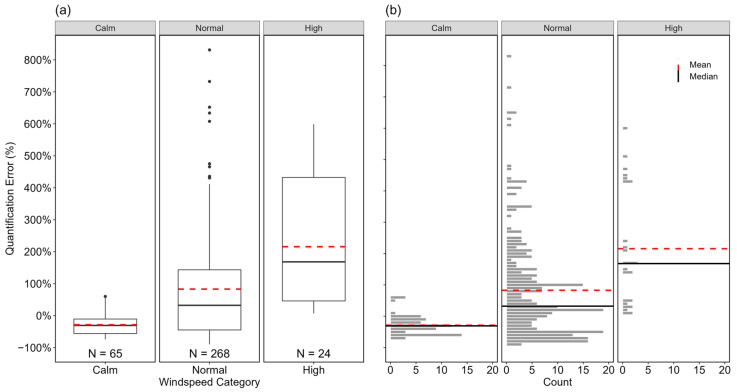
Boxplots (**a**) and distribution (**b**) of quantification errors of individual estimates based on wind speed categories (calm: 0–1 mph; normal: 2–10 mph; or high: >10 mph). The black, circular markers in (**a**) represent outliers as data points outside 1.5 times the interquartile range of the data in each group. The horizontal, black solid, and red dotted lines in both (**a**) and (**b**) indicate the median and mean of the distribution in each group, respectively. The *x*-axis represents the groups within each parameter, and the *y*-axis shows the quantification error in percentage. The numbers at the bottom of the boxplots represent the sample sizes of individual estimates within each group.

Prevailing wind speed is a categorical input parameter in the QOGI devices tested, with three defined levels: calm (0–1 mph), normal (2–10 mph), and high (>10 mph). Figure 6) show the statistically (using the Mann–Whitney U test) different (*p* < 0.05) quantification error distribution of measurements under the three windspeed categories. Results showed that the QOGI device was more accurate but likely to underestimate emissions in calm wind speed conditions with mean and median quantification errors of −29% (95% CI [−35%, −21%]) and −31%, respectively. Conversely, the wide interquartile error range along with mean and median errors of +216% (95% CI [+150%, +294%]) and 168%, respectively, indicate potential quantification challenges at high windspeed conditions (note the small sample size: *N* = 24). This is likely due to the increased turbulent and unsteady plume dispersion at high windspeed conditions, which can adversely affect the quality of plume detection. Measurements at normal wind conditions had mean and median errors of +83% (95% CI [+66%, +104%]) and 32%, respectively, which is significantly higher and lower than that at calm and high windspeed conditions, respectively, and shows that quantification became challenging as windspeed increased.

#### 3.2.4. Measurement Distance

**Figure 7:**
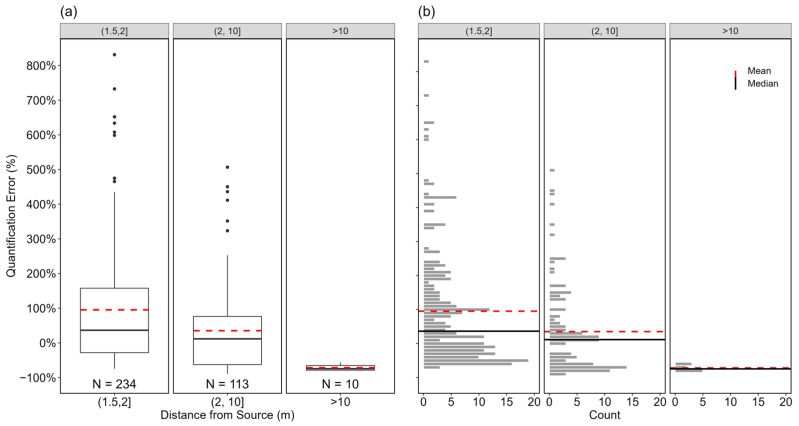
Boxplots (**a**) and distribution (**b**) of the quantification errors of individual estimates based on measurement distance ((1.5, 2] m, (2, 10] m, and >10 m). The black, circular markers in (**a**) represent outliers as data points outside 1.5 times the interquartile range of the data in each group. The horizontal, black solid, and red dotted lines in both (**a**) and (**b**) indicate the median and mean of the distribution in each group, respectively. The *x*-axis represents the groups within each parameter, and the *y*-axis shows the quantification error in percentage. The numbers at the bottom of the boxplots represent the sample sizes of individual estimates within each group.

For the QOGI tablets tested, the acceptable measurement distance from the emitting source is a function of the OGI camera lens [66]. This study used a 23 mm (24° FOV) lens which limited the measurement distance to ~1.5 m to ~16 m (5 feet to 54 feet). To investigate the impact of measurement distance on quantification performance, measurement distances were grouped into three categories: (1.5, 2] m, (2, 10] m, and > 10 m which make up 65.5%, 31.7%, and 2.8% of all the measurements as shown in Figure 7. The heavy bias towards measurements from short distances was unintentional. The choice of camera positions was dependent on space availability for the camera tripod stand, and camera perspectives adjudged to have the “best” view of the plume during measurements were typically at short distances. These constraints are likely to be seen in the field, for example, in newer production facility designs, where larger equipment groups are clustered closer than in traditional production facility designs.

The FLIR and Legacy tablets’ interface allows distance input in 0.5 m increments. Therefore, all measured distances were rounded to the closest half-meter. Due to the small sample size (*N* = 10), measurements at distances > 10 m are ignored in this discussion. As shown in Figure 7a, the mean and median errors of measurements within 2 m and (2, 10] m of the emitting source were +95% (95% CI [+75%, +118%]) and +36%, and +35% (95% CI [+14%, +62%]) and 12%, respectively. With the error distributions of measurements from both distance categories statistically different (using the Mann–Whitney U test), and the estimates from the latter distance category having lower mean and median errors with a tighter interquartile range of around 0%, the result indicates that moving the camera closer to the emitting source did not result in better quantification performance. 

This result should be taken with caution as quantification performance at measurement distances > 10 m is still unclear and will need be investigated to fully understand if the trend in increasing performance with increasing measurement distance holds for longer distances. While longer measurement distance may capture more complete plume dynamics and thus improve quantification accuracy, it can also introduce visual noise from the background or introduce adjacent components into the gas plume image, both of which adversely affect quantification performance. Additionally, small and low-pressure emissions tend to equilibrate quickly with the atmosphere as they exit the source. In these cases, the camera must be positioned closer to make the smaller plume visible in the image. For example, more than half of measurements of rates <10 slpm were performed from distances between 1.5 and 2 m.

#### 3.2.5. Diffuse vs. Point Leak Type

Figure S4 in the Supplementary Materials compared quantification performance when all other input parameters of the QOGI tablet remained the same and only the leak type (“Diffuse” or “Point”) was varied. The analysis compared the 47 estimates initially excluded from the study because the “Point” leak type instead of “Diffuse” was used for emission sources, against the 54 estimates obtained by correcting the leak type to “Diffuse”. Although the error distributions were not statistically different (*p* > 0.05: Mann–Whitney U and Kolmogorov–Smirnov tests), results showed that when “Point” was used as leak type, single estimate errors ranged from −21% to 155% with ~90% of estimates within a quantification factor of 2 compared to the error range −52% to 599% and 83% of estimates within a factor of 2 when “Diffuse” was used.

### 3.3. Observed Favorable Measurement Scenario

In actual field deployments, the emission rate is always unknown until estimated. As discussed earlier, to estimate emissions, the measurement crew intentionally chose the plume background and measurement distance, unlike the prevailing windspeed condition, which is beyond human control. Table 1 summarizes the quantification performance of the QOGI tablets under different measurement scenarios (A–E) irrespective of the prevailing windspeed condition and release rate. While a clear sky was earlier identified as the most favorable background for quantification, Table 1 shows that measurements with a plume background as equipment from distances of 1.5 to 2 m had the highest fraction of estimates within a factor of 2 (60%) with wide uncertainty. This uncertainty reduced significantly (*p* < 0.05; Kolmogorov–Smirnov tests) to 24% (scenario B with a sample size < 30) when measurement distance increased to between 2 and 10 m. For measurements with the sky as a plume background, the fraction of estimate within a factor of 2 did not statistically change (was the same at approximately 49%) as measurement distances increased from (1.5, 2] m to (2, 10] m. 

Scenario A shows the “coupling effect” of measurement conditions on quantification performance (some of which are favorable for accurate estimation) as further illustrated when the prevailing windspeed condition was factored in, see Supplementary Materials Table S5. For scenario A, under calm windspeed (0–1 mph), the fraction of estimate within a factor of 2 increased to 100% with the associated uncertainty narrowing substantially. Coincidentally, all the estimates at calm windspeed were for sources enclosed in a chamber (Supplementary Materials Figure S5). This likely improved quantification performance by limiting the rapid variability of wind conditions/plume dispersion during measurements. See Supplementary Materials Section S3.6 and Table S7 for additional analysis on quantification performance for emission points enclosed in a chamber. For scenarios D and E, under normal windspeed (2–10 mph), the fraction of estimates within a factor of 2 remained almost the same (±2%); although, the sample size for scenario D was <30. The impact of calm windspeed conditions on scenarios D and E could not be analyzed due to insufficient data; likewise, normal windspeed conditions for scenario A. The key observation from the above discussion is that the QOGI tablets tested in this study can obtain relatively accurate estimates even when all favorable measurement conditions identified do not co-exist. 

### 3.4. Quantification Precision

Quantification precision was evaluated by comparing the quantification errors of individual measurements under the same camera position and the same experiment. Ideally, given that the controlled release rate of each experiment remained approximately the same throughout all the measurements conducted for that experiment, the quantification errors of individual estimates from the same camera position were expected to be the same assuming the prevailing measurement conditions (e.g., wind speeds and background) remained constant. 

**Figure 8:**
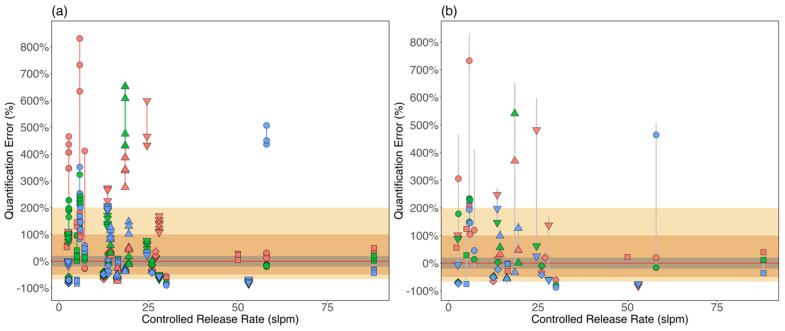
Quantification precision versus emission rate at (**a**) the camera position level and (**b**) the experiment level. The markers in (**a**) represent the errors of individual estimates/measurements. Markers of the same type and color show quantification errors of estimates obtained at the same camera positions. The whiskers connecting markers of the same color and shape show the range of quantification errors (precision range) observed at each camera position. The markers in (**b**) represent the mean error for each camera position. The whiskers connecting markers of the same shape but of different colors (mean error at camera positions) represent the precision range observed during each experiment. The gray shading represents estimates within 20% of the controlled release rate (−20% to +20% quantification error). The orange shading represents estimates within a quantification factor of two of the controlled release rates (−50% to +100% quantification error), and the yellow shading represents estimates within a factor of three of the controlled release rates (−67% to +200% quantification error).

Figure 8 shows the quantification precision at (a) the camera position level and (b) the experiment level. In Figure 8a, each marker represents a measurement/estimate. Markers of the same type represent measurements performed during the same experiment, and those with the same marker color connected by a whisker are from the same camera position during that experiment. At the camera position level, the differences between the maximum and minimum error (henceforth precision range) spanned from 2% to 439% with 75% of camera positions having precision range <50%. All nine camera positions with a precision range >100% had controlled release rates below 25 slpm; an emission rate range that also had high mean quantification errors (Figure 3 and Figure 4). This result implies that any single estimate at a camera position can differ substantially from the actual emission rate and other estimates obtained from the same camera position. Additionally, an investigation of estimates with quantification factor >5 (quantification errors > 400% or < −80%) showed that rapid variations in meteorological conditions between when measurement parameters were inputted into the QOGI tablets, and the start of actual measurements (see Supplementary Materials Section S3.6 for analysis) contributed to both the high precision range (up to 439%) and single estimate quantification error observed in this study.

Figure 8b shows the quantification precision range at the experiment level. The whiskers represent the range of individual quantification errors obtained for each experiment, regardless of camera position. The markers represent the mean quantification error for each camera position. Each whisker connecting similar markers shows the range of quantification errors for an experiment. Results show that 11 camera positions (15%) were within ±20% of controlled release rates, while 33 (45%) and 53 (73%) camera positions were within a factor of 2 (−50% to +100% error) and 3 (−67% to +200% error) of the controlled release rates, respectively. Of the 22 experiments that were quantified from three camera positions, the precision range was between 17% and 690% of the controlled release rate, indicating low measurement precision. Although the same release rate was measured throughout an experiment, measurement conditions—measurement distance, plume background, windspeed, and wind direction—varied with camera position, which likely substantially contributed to observed variation in quantification performance. In other words, this result shows the likely implication of surveyor experience on quantification performance. The different camera positions illustrate possible decisions made by surveyors of varying experience levels: the more experience a surveyor has with the OGI camera, the higher the likelihood of selecting the “best” available camera perspective to visualize a plume [33].

**Figure 9:**
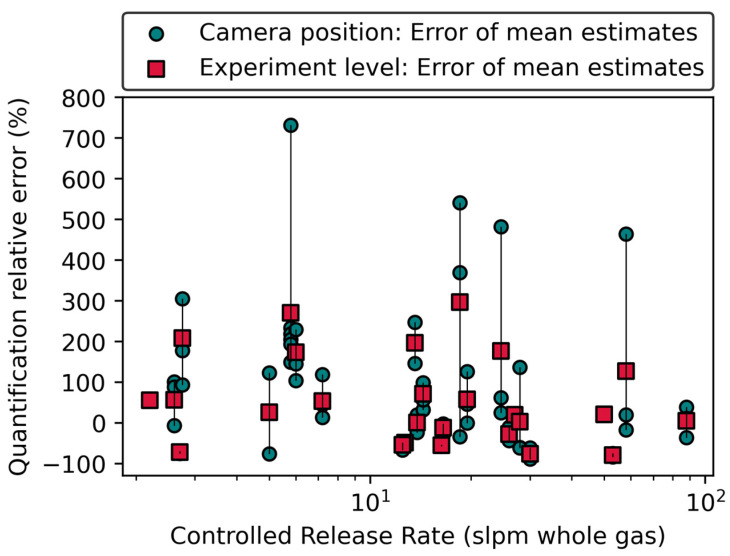
A plot of quantification relative error (linear scale) against controlled release rate (log scale). The green markers represent quantification relative errors associated with the mean of individual estimates at a camera position. The whiskers join green markers obtained during the same experiment. The red, square markers represent the mean of individual estimates obtained for each experiment.

To investigate appropriate measurement practice for more accurate estimates, Figure 9 shows quantification errors when the mean of individual estimates at either camera position (green marker) or emission/experiment (red marker) level was used. Results show that using the mean of single estimates from multiple camera positions was more accurate when compared to the mean of estimates from the same camera position. Table 2 shows that quantification error ranges narrowed from (−90%, 831%) when individual estimates were used to (−79%, 297%) when the mean of estimates from multiple camera positions under the same experiment was used. These data suggest that while careful consideration of environmental/measurement conditions during measurements is important (as shown earlier), using multiple camera positions for a leak likely generates more reliable and accurate mean estimates. Additionally, the single-estimate performance from this study was relatively more accurate (within a factor of 10) compared to the quantification performance of stationary imaging technologies and point sensors (two other methods commonly used at somewhat similar scales) which had upper limit quantification factor of ~30 for emission rate range of 0.1–1 kg ${Ch}_{4}/h$ [72,73]. These studies, however, conducted more rigorous testing consisting of simultaneous controlled releases at any time (unlike in this study), which could have affected single estimate quantification performance by the technologies tested.

While all the results discussed so far have shown the wide uncertainty on single estimates—which can significantly impact the implementation of emissions mitigation programs—some applications only prioritize quantification accuracy and the associated uncertainty when source-level estimates are aggregated at the facility or an asset level. When all individual estimates and controlled releases in this study were aggregated, the QOGI system overestimated the total controlled release rate by 43% (95% CI [+23%, +55%]) which falls within the range of values obtained for continuous monitors [72,73]. See Supplementary Materials Section S6 on simulated quantification performance in real O&G facilities.

## 4. Conclusions

This study systematically investigates the impact of release rate, plume background, and selected user input data on the quantification performance of the FLIR QL320 QOGI tool. Results indicate a wider quantification error range (−90% to +831%) than the prior study (−90% to +330%) that tested a similar QOGI tool even though the maximum rate in the current study was about an order of magnitude less than that of the prior study [65]. While our result showed a reduction in quantification error (improved accuracy and precision) as the release rate increased, a similar systematic investigation will be needed to understand quantification performance for larger rates (i.e., up to super emitter rates) which is an important emission source category. 

Study results also showed that calm windspeed (<1 mph) and viewing emissions against a clear sky background were favorable for accurate quantification. Since computational algorithms are proprietary, the cause of improved performance cannot be stated. However, less turbulent plume dispersion in calm winds provides imaging favorable for plume identification, as does viewing the plume against a clear sky where the sky’s apparent temperature is usually low, improving the thermal contrast needed for clear plume identification. Conversely, cloudy sky, vegetation on the ground, and/or backgrounds with poor ΔT were unfavorable for quantification. Although our results indicated that the distance range of 2 m to 10 m was more favorable for quantification, caution must be taken when applying this result, as with available data, we could not reliably assess quantification performance for measurement distances > 10 m. We also found that relatively accurate quantification performance can be achieved even when all favorable conditions identified above do not co-exist (Table 1 and Table S5 in the Supplementary Materials); however, there must be careful consideration in choosing measurement conditions.

The key control element for the study was the methodology applied by the OGI surveyors: The same method was used for all positions and all conditions. Controlling the measurement method removed the field crews’ experience and bias from the study design. Given that the same measurement methodology was replicable across all experiments, the wide variation (up to 690%) in quantification error as camera position changed highlights that results are highly variable based on camera position and potentially subtle changes in measurement conditions. Therefore, even though accurate emission estimates are possible even when measurement conditions are not ideal, any estimate can differ substantially from the actual emission rate. With measurement precision varying substantially as camera position/perspective changed compared to estimates obtained at the same camera position, users are likely to obtain more reliable estimates by taking measurements from more than one camera position and using the mean of all the estimates as the final value (Figure 9 and Table 2). 

The variation by camera position also implies that the experience level of a surveyor handling the OGI camera might substantially affect quantification accuracy. Hence, with the operationalization of the QOGI system in field deployment involving plume detection/visualization before quantification and results by Zimmerle et al. [33] identifying surveyor’s experience as the strongest predictor of detection rates, further studies would be needed to assess the impact of surveyor experience on quantification performance.

## Figures and Tables

**Table 1:** Table summarizes quantification performance under different scenarios (plume background and measurement distance) in this study with a sample count greater than 20. For each measurement scenario, quantification performance is illustrated with the 95% empirical confidence interval (C.I.) and the percentage of estimate within a quantification factor of 2 (−50%, 100%).

Scenario	Plume Background	Measurement Distance (m)	Sample Count	95% Empirical C.I. of Error (%)	Percentage within a Factor of 2 [−50%, 100%]
A	Equipment	(1.5, 2]	149	(−47, 639)	60
B	Equipment	(2, 10]	25	(7, 335)	24
C	Ground	(1.5, 2]	52	(−62, 241)	21
D	Sky	(1.5, 2]	33	(−68, 492)	49
E	Sky	(2, 10]	88	(−87, 432)	49

**Table 2:** Table summarizes and compares quantification performance when (1) individual estimates are used, (2) the mean estimates at camera position are used, and (3) the mean estimates for any controlled release (experiment) which includes measurement from at least 1 camera position.

Quantification Level	Percentage within a Factor of 2 [−50%, 100%]	Percentage within a Factor of 3 [−67%, 200%]	Sample Count	Error Range (%)
Individual estimates	46	75	357	(−90 to 831)
The mean of estimates at a camera position	45	72	73	(−88 to 733)
The mean of estimates at the experiment level	54	77	26	(−79, 297)

## Data Availability

The zip folder of the Supplementary Materials files contains the measurement data. The raw video files are available on request from the corresponding author.

## Supplementary Materials

The following supporting information can be downloaded at: https://www.mdpi.com/article/10.3390/s24134044/s1, Table S1: A summary of gas species, response factors, and mole fraction in the CNG releases; Figure S1: Histogram distribution of the controlled release rates from individual measurements; Figure S2: A cumulative distribution that compares controlled releases from this study (METEC data) to component-level measurements at production facilities; Figure S3: Parity chart of quantification performance between the FLIR tablet and the Legacy tablet over similar controlled release rates; Table S2: Summary statistics for Figures 4–7; Table S3: Summary of the bootstrapped 95% confidence interval for Figures 4–7; Table S4: Summary of the different measurement scenarios based on the combination of the prevailing measurement conditions. Table S5: Summary of quantification performance under different scenarios with sample count greater than 20; Figure S4: A scatter plot comparing quantification performance with leak type; Table S6: A summary of quantification performance under different scenarios for release rates and ≤ 25 slpm whole gas and quantification factor > 3; Figure S5: Figure showing illustrations of measurement of sources in an enclosed chamber; Table S7: A summary of the quantification performance of estimates of sources enclosed in a chamber. Table S8: A summary statistics of multiple linear regression analysis. Figure S6: A monte Carlo simulation of quantification errors for a field measurement campaign.
