# Supplementary material for: Methane Quantification Performance of the Quantitative Optical Gas Imaging (QOGI) System Using Single-Blind Controlled Release Assessment
Source: Sensors (Basel). 2024 Jun 21;24(13):4044. doi: 10.3390/s24134044 (PMC11244249; doi:10.3390/s24134044)
Supplement: Supplementary file 1 [file sensors-24-04044-s001.zip › sensors-3032053-supplementary.pdf]

Supplementary Information

Methane Quantification Performance of the Quantitative Optical Gas Imaging (QOGI) System Using Single-Blind Controlled Release Assessment

Chiemezie Ilonze[1], Jiayang (Lyra) Wang[2,4], Arvind P. Ravikumar[2,4], Daniel Zimmerle[4,*]

[1]Department of Mechanical Engineering, Colorado State University, Fort Collins, CO 80523
[2]Department of Petroleum and Geosystems Engineering, The University of Texas at Austin, Austin, TX 78712
[3]Energy Institute, Colorado State University, Fort Collins, CO 80524
[4]Energy Emissions Data & Modeling Lab, The University of Texas at Austin, Austin, TX 78712

[*]Corresponding author: dan.zimmerle@colostate.edu

Table of Contents

S1 Quantification measurements.......3
S1.1 Transforming measured rates using response factors.......3
S1.2 Controlled release rates.......3
S2 Quantification performance between the FLIR and the legacy tablets.......5
S3 Quantification performance analysis.......6
S3.1 Quantification error summary statistics.......6
S3.2 Confidence interval of mean quantification errors.......6
S3.3 Performance over various measurement scenarios.......7
S3.4 Comparison of quantification performance for "diffuse" vs "Point" leak types.......8
S3.5 Further investigation of quantification performance with emission rate and plume background.....8
S3.6 Analysis of quantification error "outliers" and enclosed sources.......9
S4 Multivariate Analysis.......12
S5 Monte Carlo (MC) Simulation.......12
S6 Quantification Accuracy Simulation in Active O&G Facilities.......12

List of Figures

Figure S1: Histogram distribution of the controlled release rates from individual measurements.......4

Figure S2: A cumulative distribution that compares controlled releases from this study (METEC data) to component-level measurements at production facilities...4

Figure S3: Parity chart of quantification performance between the FLIR tablet and the Legacy tablet over similar controlled release rates..5

Figure S4: A scatter plot comparing quantification performance with leak type..............................8

Figure S5: Figure showing illustrations of measurement of sources in an enclosed chamber.........10

Figure S6: A monte Carlo simulation of quantification errors for a field measurement campaign..13

List of Tables

Table S1: A summary of gas species, response factors, and mole fraction in the CNG releases.......3

Table S2: Summary statistics for Figures 4, 5, 6, and 7..6

Table S3: Summary of the bootstrapped 95% confidence interval for Figures 4, 5, 6, and 7...........6

Table S4: Summary of the different measurement scenarios based on the combination of the prevailing measurement conditions...7

Table S5: Summary of quantification performance under different scenarios with sample count greater than 20...7

Table S6: A summary of quantification performance under different scenarios for release rates and ≤ 25 slpm whole gas and quantification factor > 3..9

Table S7: A summary of the quantification performance of estimates of sources enclosed in a chamber...10

Table S8: A summary statistics of multiple linear regression analysis...11

S1 Quantification measurements

S1.1 Transforming measured rates using response factors.

One of the major advantages of the QOGI system is its ability to quantify a wide range of pure or blended gas by adjusting the measurement of a gas species without recalibrating or modifying the OGI camera or QOGI device settings. This was achieved using response factors (RF) developed for more than 400 chemical compounds. A response factor is a value that captures the relative sensitivity/response of a given pure chemical compound relative to another (propane) within the spectral window of the OGI camera. In this study, compressed natural gas (CNG) composed mostly of 88.5% methane, 8.5% ethane, 0.7% propane, 0.6% nitrogen, and 1.5% carbon dioxide were released. During the field measurement, the QOGI system was set to measure the methane mass flow rate. We subsequently transformed this methane mass flow rate using the volume-weighted RF to the whole gas flow rate. The equation is as follows:

$$\text{Adjust Mass Flowrate} = \frac{\text{Measured Rate} * \ \mathrm{RF_{methane}}}{\mathrm{RF_{equiv}}} \quad (\mathrm{S1})$$

$\mathrm{RF_{equiv}} = \ \sum_{i}^{n} y_i \, \mathrm{RF_i}$;
y_i = gas species mole fraction;
$\mathrm{RF_i}$ = response factor of gas species;
i = methane, ethane, propane, n − butane, i − pentane, hexane, carbon dioxide, nitrogen

CNG compositions during this study and their respective response factors used in Equation S1 are listed in Table S1.

Table S1: *Summary of gas species, response factors, and mole fraction in the CNG releases.*

Gas species	Response factor (RF)	Mole fraction
Methane	0.297	0.885395305
Ethane	1.082	0.084929335
Propane	1	0.007339271
n-butane	1.011	0.000649932
i-pentane	0.979	0
Hexane	1.027	0.0000649
Carbon dioxide	0	0.015295529
Nitrogen	0	0.005956317

S1.2 Controlled release rates

The controlled release rates tested in this study were unique to each experiment, ranging from 2.2 slpm to 88 slpm whole gas (2.0 slpm to 78.0 slpm of methane). A total of 26 experiments with different controlled release rates were quantified by the field crew. The distribution of the count of individual measurements with the associated controlled release rates are shown in Figure S1. The controlled release rates were not even distributed between the minimum and maximum rates. Instead, the controlled release rates concentrated towards the lower end of the range. Three

distinct groups were observed: <10 slpm, [10, 20) slpm, and ≥20 slpm. Hence, quantification errors were investigated based on these three emission rates groups in the main text.

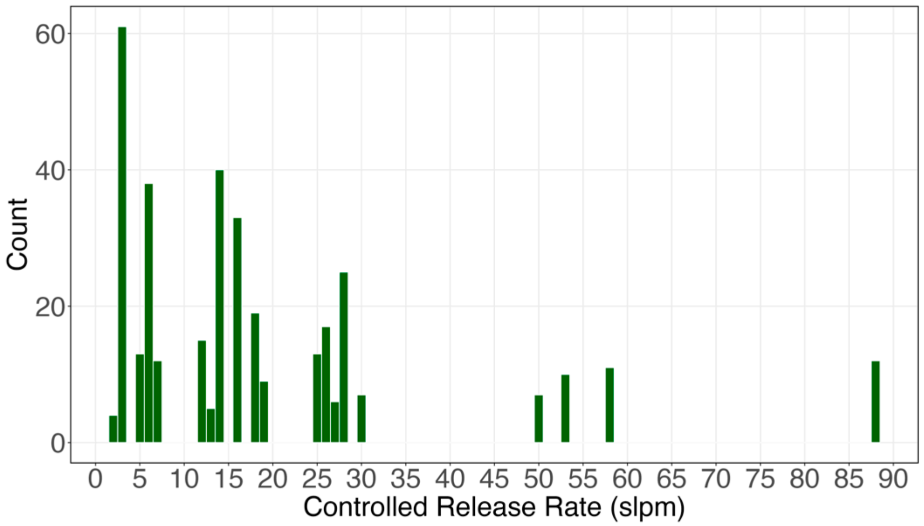

Figure S1: *Histogram distribution of the count of measurements across controlled release rates tested. The x-axis is controlled release rates (slpm), and the y-axis is the count of individual measurements.*

The emission rate range used in this study was selected given that a substantial portion of component-level emissions estimates as shown by several studies (SI Figure S2) are ≤78 slpm of methane (88 slpm whole gas/CNG).

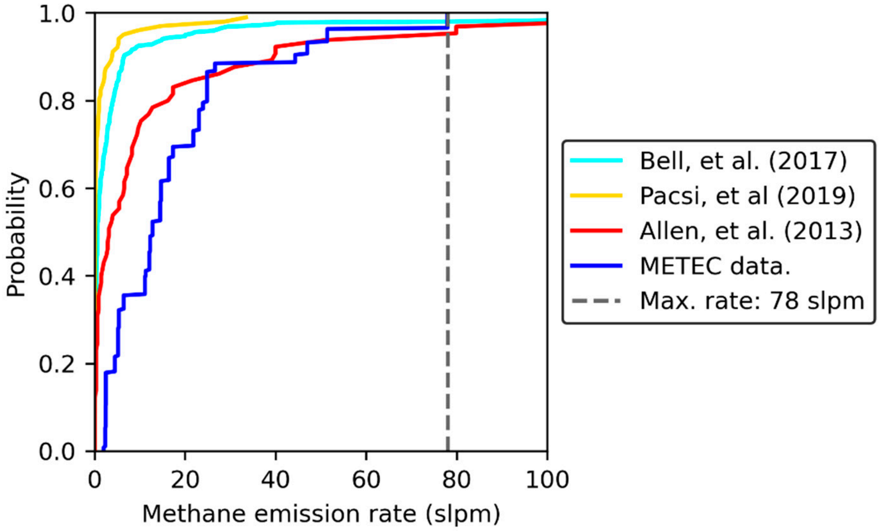

Figure S2: *A cumulative distribution that compares controlled releases from this study (METEC data) to component-level measurements at production facilities from three different studies by Bell, et al. (2017), Pacsi, et al. (2019), and Allen, et al. (2013).*

More than 90% of estimates from these studies were below the maximum rate conducted in this study (80 slpm of methane).

S2 Quantification performance between the FLIR and the legacy tablets

The FLIR tablet refers to the FLIR QL320 tablet, while the legacy tablet refers to the Providence Photonics QL320 tablet, an older version of the FLIR QL320 tablet. The legacy tablet was used when the FLIR tablets' battery was low was. Since the legacy tablet was used as a substitute for the FLIR tablet, the performance of the two tablets could not be compared. Instead, we examined the quantification performance of the two tablets when they measured similar controlled release rates. The FLIR tablet measured controlled release rates ranging from 2.2 slpm to 88 slpm of CNG/whole gas, while the legacy tablet measured controlled release rates ranging from 2.6 slpm to 5.8 slpm. We select individual measurements from the two tablets with controlled release rates ≤6 slpm. Figure S3 is a parity chart of the resulting individual measurements from the FLIR tablet (N = 37) and the legacy tablet (N = 79), and they both show overestimation bias.

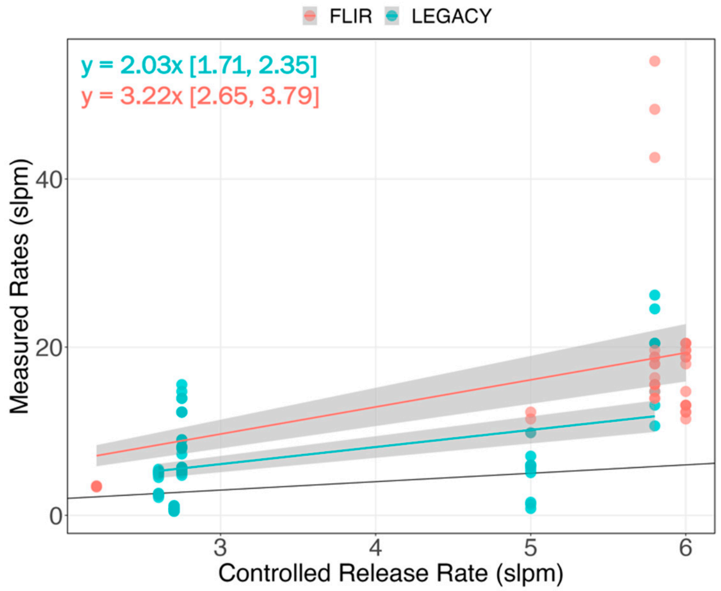

Figure S3: *Parity chart of quantification performance between the FLIR tablet (salmon color) and the Legacy tablet (blue color) over similar controlled release rates. The x-axis is the controlled release rate, and the y-axis is the measured rate. The black solid line represents a 1:1 ratio, where the measured rate equals to controlled release rate. The salmon-colored line represents the linear regression of the FLIR tablet, and the blue-colored line represents the linear regression of the Legacy tablet. The gray area represents a 95% confidence interval of the linear regression coefficient.*

A linear regression analysis of measurements obtained with the FLIR tablet showed a linear regression coefficient of 1.27 (95% CI [1.11, 1.42]), which is almost equal to the regression result (1.27 (95% CI [1.13, 1.40]) obtained using all the measurement data (FLIR + Legacy) as shown in Figure 3a in the main text. Consequently, using the legacy tablet did not introduce a statistically significant difference in the study results.

S3 Quantification performance analysis

S3.1 Quantification error summary statistics

Table S2 shows the summary statistics for Figures 4, 5, 6, and 7 in the main text. Note that quantification errors are presented as percentages in the graphs.

Table S2*: Quantification error summary statistics for each quantification parameter.*

	Min	25% Quartile	Median	Mean	75% Quartile	Max	Standard Dev
Controlled release rates (slpm)							
<10	-0.84	-0.13	0.99	1.19	1.98	8.31	1.60
[10, 20)	-0.74	-0.48	-0.03	0.65	1.27	6.52	1.62
≥20	-0.90	-0.59	-0.15	0.22	0.28	5.99	1.34
Quantification background							
Equipment	-0.52	-0.06	0.84	1.22	1.98	8.31	1.72
Ground	-0.68	-0.59	0.89	0.68	1.38	2.41	1.04
Sky	-0.90	-0.67	-0.44	0.05	0.25	5.99	1.30
Wind speed							
Calm	-0.74	-0.56	-0.31	-0.29	-0.11	0.60	0.31
Normal	-0.90	-0.45	0.32	0.83	1.43	8.31	1.60
High	0.07	0.46	1.68	2.16	4.32	5.99	1.86
Distance (m)							
(1.5,2]	-0.74	-0.28	0.36	0.95	1.58	8.31	1.68
(2,10]	-0.90	-0.63	0.12	0.35	0.76	5.07	1.27
>10	-0.80	-0.79	-0.75	-0.72	-0.65	-0.56	0.09

S3.2 Confidence interval of mean quantification errors

Table S3 *Summarizes the bootstrapped 95% confidence interval for Figures 4, 5, 6, and 7 in the main text.*

	Median	Mean	95% CI Lower	95% CI Upper
Controlled release rates (slpm)				
<10	0.99	1.19	0.94	1.50
[10, 20)	-0.03	0.65	0.40	0.99
≥20	-0.15	0.22	0.003	0.53
Quantification background				
Equipment	0.84	1.22	0.98	1.50
Ground	0.89	0.68	0.40	0.97
Sky	-0.44	0.05	-0.13	0.32
Wind speed				
Calm	-0.31	-0.29	-0.35	-0.21
Normal	0.32	0.83	0.66	1.04
High	1.68	2.16	1.50	2.94
Distance (m)				
(1.5,2]	0.36	0.95	0.75	1.18
(2,10]	0.12	0.35	0.14	0.62

>10	-0.75	-0.72	-0.76	-0.66

S3.3 Performance over various measurement scenarios

Table S4 *summarizes the different measurement scenarios based on the combination of the prevailing measurement conditions while showing the fraction of measurements within a factor of 2.*

Plume background	Wind speed	Emission rate (slpm)	Distance (m)	Count	95% CI of Error (%)	Fraction within a factor of 2 (%)
Equipment	Calm	[10, 20)	(1.5, 2]	23	(-38.1, 6.4)	100.0
Equipment	Calm	>20	(1.5, 2]	17	(-43.3, -0.5)	100.0
Equipment	Normal	<10	(1.5, 2]	49	(-8.1, 712.7)	38.8
Equipment	Normal	<10	(2, 10]	8	(90.8, 346.6)	12.5
Equipment	Normal	[10, 20)	(1.5, 2]	44	(-50.3, 648.7)	31.8
Equipment	Normal	[10, 20)	(2, 10]	3	(102.9, 146.8)	0.0
Equipment	Normal	>20	(1.5, 2]	13	(7.9, 33.8)	100.0
Equipment	High	<10	(2, 10]	9	(139.9, 235.8)	0.0
Equipment	High	[10, 20)	(1.5, 2]	3	(42.9, 50.9)	100.0
Equipment	High	[10, 20)	(2, 10]	5	(6.7, 30.5)	100.0
Ground	Calm	>20	(1.5, 2]	17	(-65.5, -51.5)	0.0
Ground	Normal	<10	(1.5, 2]	15	(95.7, 241.0)	6.7
Ground	Normal	[10, 20)	(1.5, 2]	12	(22.5, 124.2)	83.3
Ground	Normal	>20	(1.5, 2]	8	(108.0, 166.3)	0.0
Sky	Calm	<10	(1.5, 2]	4	(52.8. 59.7)	100.0
Sky	Calm	[10, 20)	(1.5, 2]	4	(-73.8, -26.6)	50.0
Sky	Normal	<10	(2, 10]	43	(-82.4, 144.7)	44.2
Sky	Normal	[10, 20)	(1.5, 2]	13	(-57.2, -49.2)	15.4
Sky	Normal	[10, 20)	(2, 10]	14	(-66.6, -40.0)	21.4
Sky	Normal	>20	(1.5, 2]	8	(-20.2, 48.8)	100.0
Sky	Normal	>20	(2, 10]	28	(-87.9, 69.6)	75.0
Sky	Normal	>20	>10	10	(-79.9, -57.6)	0.0
Sky	High	>20	(1.5, 2]	4	(432.3, 588.6)	0.0
Sky	High	>20	(2, 10]	3	(436.9, 503.9	0.0

Table S5 *summarizes quantification performance under different scenarios (plume background, measurement distance, and windspeed condition) in this study with a sample count greater than 20. For each scenario, quantification performance is illustrated with the 95% empirical confidence interval (C.I.) and the percentage of estimate within a quantification factor of 2 (-50%, 100%).*

Plume Background	Measurement Distance (m)	Windspeed Category	Sample Count	95% Empirical C.I. of Error (%)	Percentage within a Factor of 2 [-50%, 100%]
Equipment	(1.5, 2]	Calm	40	(-43, 4)	100
Equipment	(1.5, 2]	Normal	106	(-49, 652)	43
Ground	(1.5, 2]	Normal	35	(29, 241)	31
Sky	(1.5, 2]	Normal	21	(-57, 49)	48
Sky	(2, 10]	Normal	85	(-87, 126)	51

S3.4 Comparison of quantification performance for “Diffuse” vs “Point” leak types

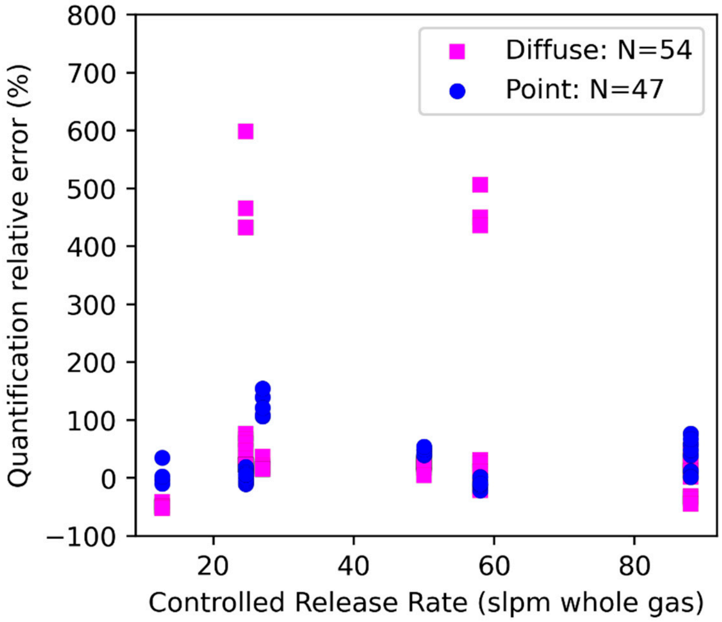

Figure S4*: A scatterplot comparing quantification performance when leak type “Diffuse” or “Point” is selected as an input parameter into the QOGI tablet during a measurement. The plot compares the 47 estimates initially excluded from the analysis because "Point" was used as the leak type instead of "Diffuse" as defined in the QOGI user manual, against 54 estimates of the same controlled release using “Diffuse” as the leak type.*

S3.5 Further investigation of quantification performance with emission rate and plume background.

Figure 3 in the main text shows that the majority (80%: 73 of 91) of all estimates with quantification relative errors < -66.67% or > 200% (quantification factor > 3) were obtained when the controlled release rate was ≤ 25 slpm whole gas. When all controlled releases with rates ≤ 25 slpm (N = 262) were considered, about 28% (73 of 262) of estimates had quantification relative errors < -66.67% or > 200% compared to 18% (17 of 95) for rates > 25 slpm of whole gas. This illustrates the observed improvement of quantification performance with emission rate. Table S6 shows which measurement scenario had the highest and lowest percentage of estimates of controlled release less than 25 slpm CNG with quantification factor > 3.

Table S6 *summarizes quantification performance under different scenarios (windspeed category, plume background, and measurement distance) for release rates ≤* 25 slpm whole gas and quantification factor > 3 (quantification relative error < -66.7%, quantification relative error > 200%).

Windspeed Category	Plume Background	Measurement Distance (m)	Sample Count	95% Empirical C.I. of Error (%)	As a percentage of all emission rates tested & quantification errors‡
Normal	Equipment	(1.5, 2]	35	(206, 747)	33%
Normal	Equipment	(2, 10]	5	(253, 349)	46%
Normal	Ground	(1.5, 2]	8	(203, 241)	23%
Normal	Sky	(2, 10]	17	(-83, 220)	20%
High	Equipment	(2, 10]	3	(211, 238)	21%
High	Sky	(1.5, 2]	4	(432, 589)	100%
Calm	Sky	(1.5, 2]	1	(-74, -74)	13%

‡ For estimates obtained under a specified wind category, viewed against a given plume background and conducted from a given measurement distance, this is calculated as the percentage of estimates of controlled release ≤ 25 with quantification relative errors < -66.67% or > 200% (quantification factor > 3). For example, 33% of estimates at normal windspeed with equipment as background and measurement distance between 1.5m and 2m which was obtained when quantifying controlled releases of rates ≤ 25 slpm had quantification relative errors < -66.67% or > 200%.

S3.6 Analysis of quantification error "outliers" and enclosed sources.

For estimates (34 of 357) with quantification errors > 400% or <-80%, we investigated the impact of the variation of wind speed condition inputted into the QOGI tablets before measurement and the prevailing wind condition during measurement. For this analysis, we used the measurement timestamp of the 34 estimates to find the prevailing wind condition at the time of measurement using the wind data from METEC's meteorological station (~ 24ft in height). The station also records other atmospheric data including relative humidity, temperature, etc. at a frequency of 1Hz. For the 34 of 357 individual estimates with quantification errors > 400% or < -80% (> quantification factor of 5) obtained from 13 camera positions as shown in Figure 3(a), we found that 7 of the 13 (54%) also had other estimates within a factor < 5 and even up to a factor of 2 in some cases. Given the time lag between when measurement parameters were inputted into the QOGI tablets and the start of actual measurements, we found that for 22 of the 34 (65%) estimates, the wind category at the time of measurement differed from what was initially inputted into the tablet due to rapid variability of wind condition (normal ↔ high). Also, results showed that 73% of the estimates from those 7 camera positions which also had other estimates within quantification factor < 5, also experienced similar variation of wind condition during measurement. These rapid meteorological variations likely introduced substantial errors in quantification estimates given that the algorithm of the tablets depends on the input parameters to perform quantification. Of the remaining 6 of 13 camera positions, 2 camera positions were responsible for all estimates under high windspeed conditions with leak type "diffuse thereby illustrating the difficulty associated with accurate emissions quantification under that scenario. When estimates

(N=3) from the first camera position in the study which is responsible for the highest quantification errors in the study (634%, 732%, and 831%) were excluded, 11 of the 12 (92%) estimates from the remaining 3 camera positions had different wind condition at the time of measurement compared to the values in the tablet. By excluding the estimates from the first camera position, the individual relative errors from the study ranged from -90% to +652%.

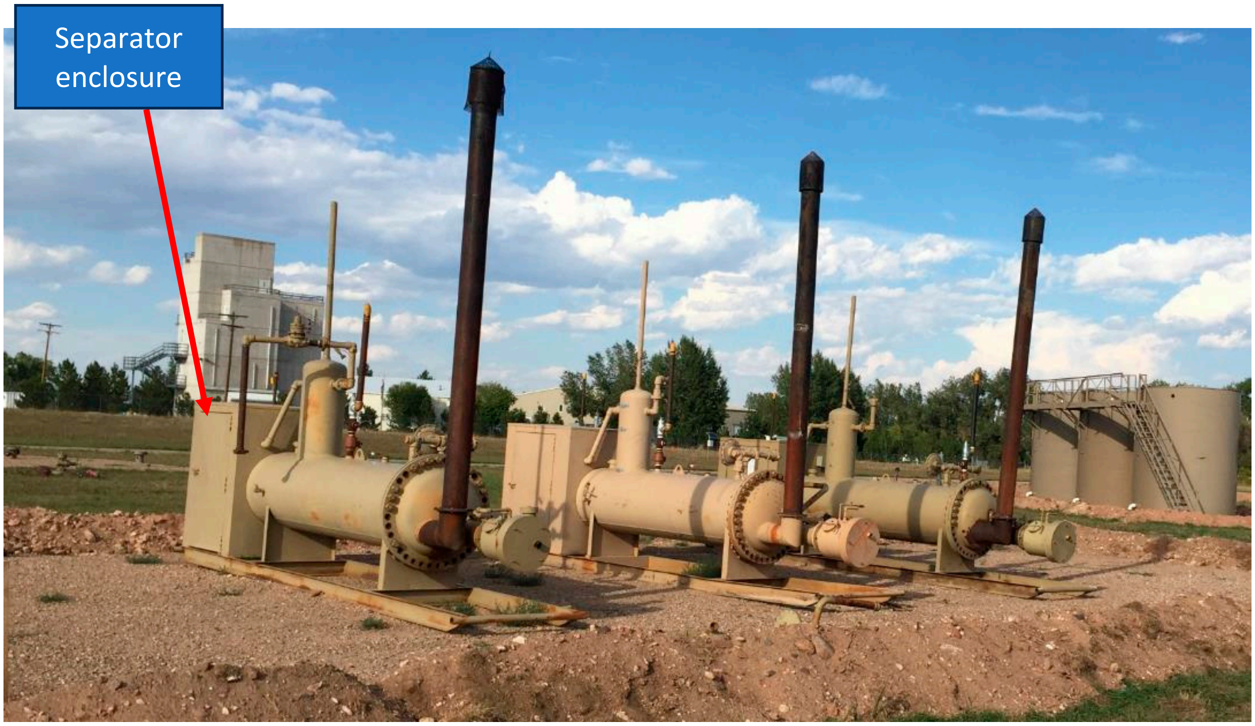

Figure S5: *The figure shows a group of separators at METEC with an arrow showing an enclosure housing some emission points used in the study.*

To evaluate the influence of chambers/enclosures on quantification performance, the field crew documented if a source was enclosed or open-air during measurements. There were 47 estimates of sources enclosed within a separator chamber (plume background as Equipment) as indicated in Figure S5 above. Table S7 summarizes the quantification performance based on various measurement conditions encountered. We found that all estimates at calm windspeed conditions from a measurement distance between 1.5m to 2m were within a factor of 2. This is likely because under calm wind condition, plume dispersion is more stable, and the chamber limits the variability of the plume dispersion profile which can improve quantification performance.

Table S7 *summarizes the quantification performance of estimates of sources enclosed in a chamber (plume background is equipment) under different measurement scenarios encountered during their measurement.*

Plume background	Wind speed	Distance (m)	Count	95% CI of Error (%)	Fraction within a factor of 2 (%)
Equipment	Calm	(1.5, 2]	40	(-43.3, 4.3)	100.0
Equipment	Normal	(1.5, 2]	7	(82.6, 636.5)	12.7

S4 Multivariable analysis

We conducted multiple linear regression analysis to evaluate the relative importance of different parameters for quantification errors. We selected controlled release rate, measuring distance, quantification background, and wind speed as input variables. Figure S3 shows the summary statistics of the regression.

```
Call:
lm(formula = Error ~ 0 + EmissionSize + Distance + Background +
    WindSpeed, data = dfIndMulti)

Residuals:
    Min      1Q  Median      3Q     Max
-2.3363 -0.8893 -0.1343  0.3592  6.4049

Coefficients:
                    Estimate Std. Error t value Pr(>|t|)
EmissionSize≥ 20     -1.7616     0.2387  -7.379 1.18e-12 ***
EmissionSize<10      -1.3259     0.2699  -4.912 1.39e-06 ***
EmissionSize10 - 20  -1.9300     0.2676  -7.212 3.47e-12 ***
Distance(1.5,2]       0.9196     0.2130   4.317 2.07e-05 ***
Distance>10          -0.4733     0.4475  -1.058   0.2910
BackgroundEquipment   0.7955     0.2021   3.936   0.0001 ***
BackgroundGround      0.4146     0.2611   1.588   0.1132
WindSpeedHigh         2.9780     0.3382   8.805  < 2e-16 ***
WindSpeedNormal       1.5183     0.2000   7.590 2.96e-13 ***
---
Signif. codes:  0 ‘***’ 0.001 ‘**’ 0.01 ‘*’ 0.05 ‘.’ 0.1 ‘ ’ 1

Residual standard error: 1.297 on 348 degrees of freedom
Multiple R-squared:  0.4545,    Adjusted R-squared:  0.4404
F-statistic: 32.21 on 9 and 348 DF,  p-value: < 2.2e-16
```

All variables were treated as categorical variables following the binning in Figure 2. Categories with the mean quantification error closest to 0% were used as the reference level for categorical variables in the regression analysis – "≥20" for controlled release rate, "(2, 10]" for measuring distance, "Sky" for quantification background and, "Calm" for wind speed. The analysis showed that (1.5, 2] measuring distance, equipment background, high wind speed, and normal wind speed had a statistically significant impact on quantification error ($p < 0.05$). When all other parameters were held constant, the mean quantification error from measurements taken from (1.5, 2]m was 92% (regression coefficient of 0.92) higher than that of the (2, 10]m measurement distance. Similarly, the mean quantification error for measurements taken with equipment as background was 80% higher than that of the sky background. Both high and normal wind speed conditions introduced positive quantification error bias compared to calm wind speed. The mean quantification error from measurements quantified at high and normal wind speed conditions were 298% and 152% higher than that of calm wind speed, respectively.

Table S8: *Summary statistics of multiple linear regression analysis using RStudio.*

Variable	Coefficient	p-Value

Intercept	-1.76	<0.001
Controlled release rate - <10	0.44	0.02
Controlled release rate - 10 - 20	-0.17	0.37
Measuring distance - (1.5, 2]	0.92	<0.001
Measuring distance - >10	-0.47	0.29
Quantification background - Equipment	0.80	<0.001
Quantification background - Ground	0.41	0.11
Wind speed - High	2.98	<0.001
Wind speed - Normal	1.52	<0.001

S5 Monte Carlo (MC) Simulation

The MC analysis was used to simulate the likely performance of the QOGI tool while quantifying leaks of size within the tested range (2slpm and 90 slpm of CNG/whole gas) in this study during field deployments. The analysis assumed that the source-level estimates (measured using a hi-flow sampler) from a field measurement study as the "true" rates of the leaks. The MC simulations applied the relation x*e + x to generate the likely estimate of a leak of size x using the relative error e obtained in this study. The result from the analysis is shown in Figure 4 of the main paper and the process below describes how the analysis was performed.

- The selected field measurement data was divided into two groups: 2 – 25 slpm and 25 – 90 slpm along with the relative quantification error distributions associated with each group as obtained in this study.
- A random sample (x) was picked from the selected field measurement data without replacement.
- For each x, an error (e) from the relative error distribution associated with the emission rate group which x falls within was randomly picked. The relation x*e + x was applied to calculate a quantification estimate.
- For any given x, the process above was repeated 10,000 times generating 10,000 estimates of x for all x in the measurement data. This generates an array of size N by 10,000 where N represents the count of the selected field measurement data.
- All rows were summed to generate 10,000 aggregated estimates. The selected field measurement data was also aggregated and used to compute 10,000 relative errors.
- The mean of the 10,000 relative errors was assessed along with the 95% empirical confidence interval (CI) evaluated as 2.5%, and 97.5% percentiles of the 10,000 relative errors.

S6 Quantification Accuracy Simulation in Active O&G Facilities

While results in the main text have shown the wide uncertainty on single estimates which can significantly impact emissions mitigation programs, some applications only prioritize quantification accuracy and the associated uncertainty when source-level estimates are aggregated at the facility or asset level. When all individual estimates and controlled releases in this study were aggregated, the QOGI system overestimated the total controlled release rate by

43% (95% CI [+23%, +55%]). To evaluate the potential quantification performance of the QOGI system during field deployments, we performed an MC analysis simulating facility-level quantification with its associated uncertainty. The analysis used the error distribution from this study and the component-level measurement data from Zimmerle et al [22]. Measurement data from 150 facilities with rates within the tested range in this study (2slpm and 90 slpm) were considered as the true rates in the MC simulation with the number of leaks per facility ranging from 1 to 58 (mean of 6). Results from the MC simulations are shown in Figure 4 below.

Figure S6a shows the MC simulation (SI section S5) analysis of the facility-level quantification error (with its associated uncertainty) for each of the 150 facilities from the field study [22]. Results indicated that while on the mean, the aggregated estimates were within a quantification factor of 2 (-50% to 100%), the upper bound of the associated uncertainties (empirical 95% CI on the mean) was within a quantification factor of ~7. Unsurprisingly, the uncertainties became narrower as the count of measured emissions per facility increased which is consistent with the AMFC study and that of the Concawe air quality OGI ad-hoc group which identified a similar trend for Method 21 correlations over many leaks [63]. To highlight the likely impact of quantification uncertainties on regulatory methane reduction programs like the IRA, the study performed an MC simulation assessing the mean error and the associated uncertainty for all 150 facilities aggregated. Figure S6(b) shows a cumulative distribution function (CDF) of the errors from the MC simulation with a mean error of +22.1% (empirical 95% CI of +13.2% to +31.4%). Assuming the simulated emissions from all the 150 facilities aggregated were above the threshold set by IRA and an operator owned all of them, the methane fee payment could vary from [$1.8/hr to $4.2/hr] at $900/ CH4 mt to [$3.0/hr to $7.1/hr] at $1500/ CH4 mt which could have substantial financial implications on operator [8].

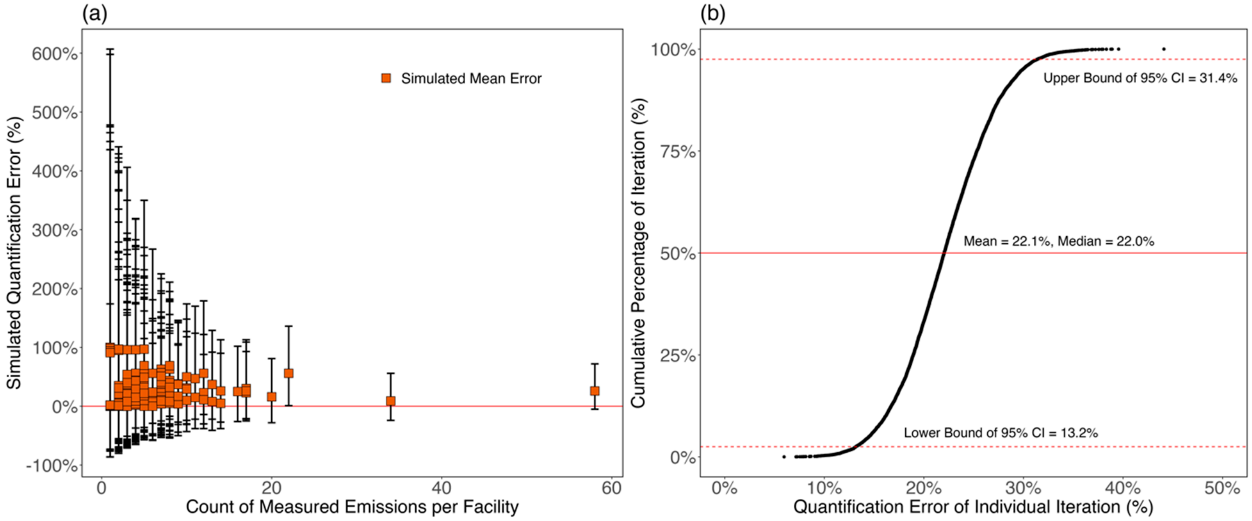

Figure S6: *Monte-Carlo simulated quantification error by (a) count of measured emissions per facility and (b) total emissions from 150 facilities. In (a), the x-axis is the count of measured emissions on each facility and the y-axis is the simulated quantification error. The orange square represents the mean of quantification errors from the 10,000 Monte-Carlo simulations for each facility. The error bars represent 95% confidence intervals of the mean. In (b), the x-axis is rank ordered (CDF) quantification error of individual iteration, which represents the quantification error*

of total simulated emissions from 150 facilities. The y-axis is the cumulative percentage of the count of iterations. The horizontal red solid line is the median of quantification error, and the horizontal red dashed lines are the 95% confidence interval of quantification error.
